# Pharmacologic targeting ERK1/2 attenuates the development and progression of hyperuricemic nephropathy in rats

**DOI:** 10.18632/oncotarget.16995

**Published:** 2017-04-10

**Authors:** Na Liu, Liuqing Xu, Yingfeng Shi, Lu Fang, Hongwei Gu, Hongrui Wang, Xiaoqiang Ding, Shougang Zhuang

**Affiliations:** ^1^ Department of Nephrology, Shanghai East Hospital, Tongji University School of Medicine, Shanghai 200120, China; ^2^ Division of Nephrology, Zhongshan Hospital, Fudan University, Shanghai 200032, China; ^3^ Department of Medicine, Rhode Island Hospital and Brown University School of Medicine, Providence, RI 02903, USA

**Keywords:** hyperuricemic nephropathy, ERK1/2, TGF-β/Smad signaling pathway, urate transporters, inflammation

## Abstract

The pathogenesis of hyperuricemia-induced chronic kidney disease is largely unknown. In this study, we investigated whether extracellular signal–regulated kinases1/2 (ERK1/2) would contribute to the development of hyperuricemic nephropathy (HN). In a rat model of HN induced by feeding mixture of adenine and potassium oxonate, increased ERK1/2 phosphorylation and severe glomerular sclerosis and renal interstitial fibrosis were evident, in parallel with diminished levels of renal function and increased urine microalbumin excretion. Administration of U0126, which is a selective inhibitor of the ERK1/2 pathway, improved renal function, decreased urine microalbumin and inhibited activation of renal interstitial fibroblasts as well as accumulation of extracellular proteins. U0126 also inhibited hyperuricemia-induced expression of multiple profibrogenic cytokines/chemokines and infiltration of macrophages in the kidney. Furthermore, U0126 treatment suppressed xanthine oxidase, which mediates uric acid production. It also reduced expression of the urate anion exchanger 1, which promotes reabsorption of uric acid, and preserved expression of organic anion transporters 1 and 3, which accelerate uric acid excretion in the kidney of hyperuricemic rats. Finally, U0126 inhibited phosphorylation of Smad3, a key mediator in transforming growth factor (TGF-β) signaling. In cultured renal interstitial fibroblasts, inhibition of ERK1/2 activation by siRNA suppressed uric acid-induced activation of renal interstitial fibroblasts. Collectively, pharmacologic targeting of ERK1/2 can alleviate HN by suppressing TGF-β signaling, reducing inflammation responses, and inhibiting the molecular processes associated with elevation of blood uric acid levels in the body. Thus, ERK1/2 inhibition may be a potential approach for the prevention and treatment of hyperuricemic nephropathy.

## INTRODUCTION

The prevalence of chronic kidney disease (CKD) continues to elevate worldwide [1]. Patients with CKD will gradually progress into the end stage of renal disease (ESRD) [2]. Thus, it is imperative to investigate the risk factors that promote CKD progression. Multiple observational studies have indicated that hyperuricemia is a risk factor for CKD [3–5]. In addition, results from animal studies have also shown that elevation of serum uric acid plays an important role in the onset and progression of CKD [6, 7]. Therefore, it is important to investigate the molecular mechanism that results in hyperuricemia [8–10] and subsequent hyperuricemic nephropathy.

Uric acid is an end product of purine metabolism. Hyperuricemia occurs as a result of increased serum uric acid production and decreased uric acid excretion in urine. In the kidney, urate is filtered by the glomerulus and subsequently reabsorbed by the proximal tubular; normal minimum excretion of uric acid is 10% [11]. In some pathologic circumstances, elevated cell turnover leads to increased extracellular levels of adenosine, inosine and guanosine [12]. For instance, chemotherapeutic treatments for some diseases such as leukemia or lymphoma, commonly increase the excretion of uric acid due to promotion of nucleic acid metabolism, leading to obstruction of renal tubules and induction of acute kidney injury [13]. These nucleobases are further degraded to hypoxanthine and xanthine, which are the substrates for the enzyme xanthine oxidase (XO), a key enzyme in the formation of uric acid [11]. Urate transport depends on specific and selective transporters. It is generally believed that the human urate transporter, urate anion exchanger 1(URAT1) (encoded by the*SLC22A12* gene), facilitates uric acid reabsorption in the proximal convoluted tubules [14]. Whereas, both OAT1 (encoded by the *SLC22A6*) and OAT3 (encoded by the *SLC22A8*), two urate excretion transporters, promote excretion of urate from the body [15, 16]. Therefore, increased expression and activation of URAT1 and decreased expression and activation of OAT1 and OAT3 will lead to accumulation of uric acid in the body.

Mechanisms of renal injury induced by hyperuricemia remain obscure. Uric acid induced direct obstruction in tubules and indirect damage may play a pivotal role in the progression of hyperuricemic nephropathy. This was made evident by the observation of the formation and deposition of uric acid crystals, surrounded by monocytes/macrophages in the kidneys of hyperuricemic rats [17]. Hyperuricemic rats also displayed severe tubular damage and significantly enhanced expression of multiple cytokines, such as (transforming growth factor-β1 (TGF-β1), interluckin-1 (IL-1), interlukine-6 (IL-6), and tumor necrotic factor-α (TNF-α) and chemokines, monocyte chemoattractant protein-1 (MCP-1) and RANTES [18, 19]. However, numerous studies have indicated that development and progression of hyperuricemic nephropathy can occur through a mechanism independent of the formation of acid crystals [9]. For example, mild hyperuricemia can cause hypertension and renal injury in the rat via a crystal-independent mechanism that is associated with stimulation of the renin-angiotensin system and inhibition of neuronal NO synthase [20]. In addition, uric acid can induce tyrosine kinase receptor activation, oxidative stress, tubular epithelial cell transition, renal interstitial fibroblast activation and vascular endothelial damage [9, 21–24].

Recently, we have demonstrated that inhibition of epidermal growth factor receptor (EGFR) can alleviate the development of HN [9]. EGFR is one of tyrosine kinase receptors that initiates activation of multiple signaling pathways, including extracellular signal–regulated kinases1/2 (ERK1/2). A number of studies demonstrated that activation of the ERK1/2 pathway contributes to acute kidney injury [25, 26] and is associated with pathogenesis of chronic allograft nephropathy in an animal model [27]. *In vitro* studies have also shown that activation of the ERK1/2 pathway promotes proliferation of uric acid-induced mesangial cells [28]. Mechanistically, ERK1/2 has been reported to directly induce phosphorylation of Smad3, a key signaling molecule in the TGF-β signaling pathway [29]. Conversely, the TGF-β pathway can also induce activation of the ERK1/2 pathway [30, 31]. These studies clearly indicate that ERK1/2 are coupled to the signaling pathway that induces chronic renal changes. However, it remains unclear whether inhibition of the ERK1/2 pathway is capable of halting or slowing the progression of HN.

The purpose of this study was to examine whether ERK1/2 signaling would be activated in the pathogenesis of HN in a rat model and whether pharmacological inhibition of ERK1/2 signaling would have impact on this process.

## RESULTS

### Silencing of ERK1/2 blocks uric acid-induced activation of cultured renal interstitial fibroblasts

**Figure 1:**
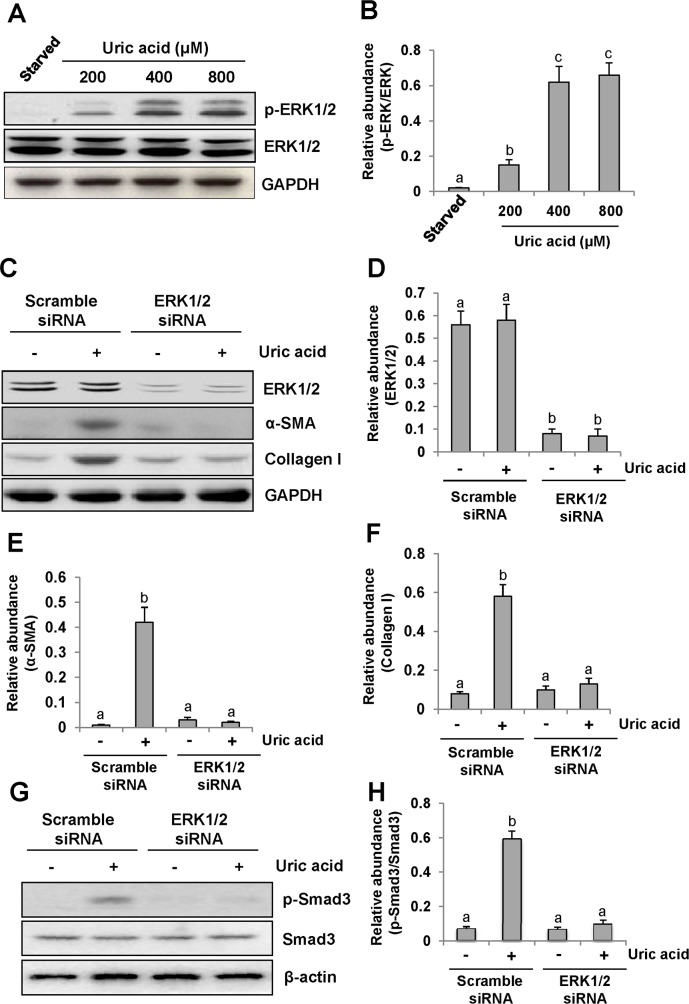
Uric acid dose-dependently induces ERK1/2 phosphorylation in cultured renal interstitial fibroblasts and the effect of ERK1/2 silencing on the activation of renal interstitial fibroblasts Cultured NRK-49F cells were starved for 24h and then exposed to various concentrations of uric acid (0-800 μM) for 36h. Then, cell lysates were subjected to immunoblot analysis with antibodies against p-ERK1/2, ERK1/2 or glyceraldehyde 3-phosphate dehydrogenase (GAPDH) **(A and B)**. The level of p-ERK1/2 was quantified by densitometry and normalized with ERK1/2(B). Serum starved NRK-49F cells were transfected with siRNA targeting ERK1/2 or scrambled siRNA and then exposed to uric acid (800 μM) for 36h **(C)**. Cells lysates were subjected to immunoblot analysis with antibodies against ERK1/2, α-SMA, Collagen-I or GAPDH. Expression levels of ERK1/2, α-SMA or Collagen-I were quantified by densitometry and normalized with GAPDH **(D-F)**. In addition, immunoblot analysis was also performed with antibodies against p-Smad3, Smad3 or β-actin and p-Smad3was quantified by densitometry and normalized with Smad3 **(G and H)**. Values are means±SDs of at least three independent experiments. Bars with different letters (a-c) for each molecule are significantly different from one another (*P*<0.05).

Recently, we reported that uric acid exposure induced activation of renal interstitial fibroblasts, which was blocked by treatment with U0126, a selective inhibitor of the ERK1/2 pathway [9]. To verify this, we further examined phosphorylation (activation) of ERK1/2 in response to various doses of uric acid and the effect of siRNA mediated silencing ERK1/2 on renal fibroblast activation. As shown in Figure 1A–1B, exposure of NRK-49F to uric acid at 200-800 μM resulted in increased phosphorylation (activation of ERK1/2) in a dose dependent manner, with the maximum induction at 800 μM. In parallel, this dose of uric acid also induced activation of renal interstitial fibroblasts as evidenced by increased expression of both α-SMA and collagen 1. Transfection of ERK1/2 siRNA (small interference RNA) reduced ERK1/2 and suppressed α-SMA and collagen 1 expression of renal fibroblasts in response to uric acid (Figure 1C–1F). Since Smad3 is a key signaling molecule in TGF-β signaling pathway [30], we examined the effect of siRNA-mediated silencing of ERK1/2 on its activation in cultured fibroblasts exposed to uric acid *in vitro*. Our results demonstrated that downregulation of ERK1/2 by siRNA significantly suppressed uric acid-induced phosphorylation of Smad3 in renal interstitial fibroblasts (Figure 1G–1H), indicating that specific inhibition of ERK1/2 kinases with siRNA was also effective in reducing activation of the TGF-β/Smad3 signaling pathway. Therefore, we confirmed that ERK1/2 played an important role in mediating uric acid-induced activation of renal interstitial fibroblasts.

### Time dependent induction of renal fibrosis and activation of ERK1/2 in a rat model of hyperuricemic nephropathy

**Figure 2:**
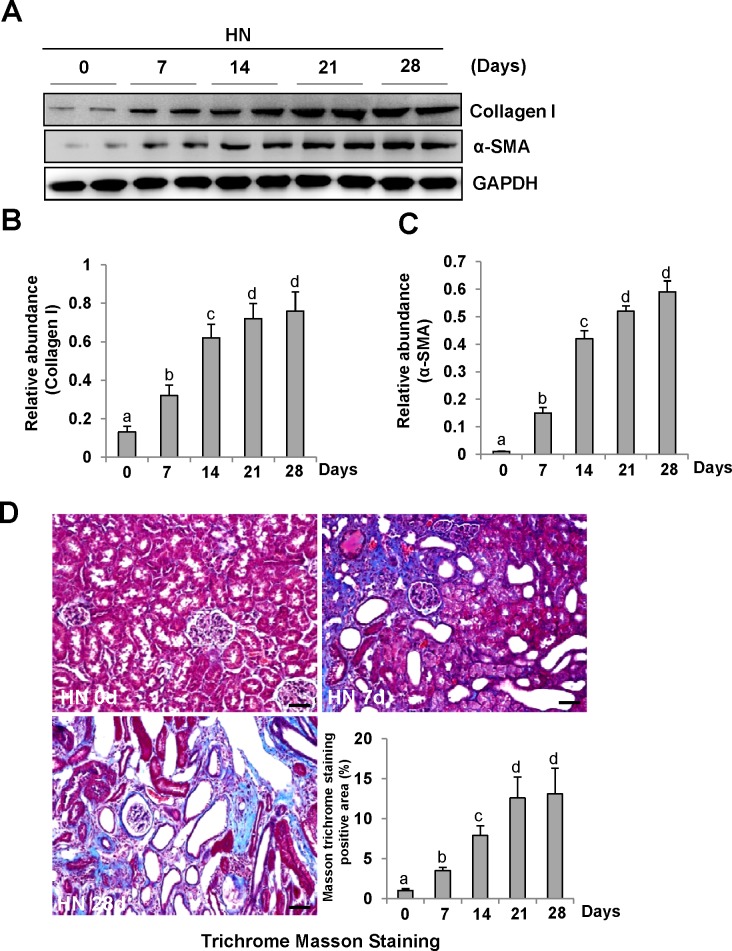
Activation of renal interstitial fibroblasts in the kidney of hyperuricemic rats in a time-dependent manner **(A)** The kidneys were taken for immunoblot analysis of Collagen-I, α-SMA or glyceraldehyde 3-phosphate dehydrogenase(GAPDH); the representative results with two samples are shown. **(B and C)** Expression levels of Collagen I or α-SMA were quantified by densitometry and normalized with GAPDH. (C) Photomicrographs illustrating Masson trichrome staining of kidney after feeding with adenine and potassium oxonate at different times. **(D)** The graph shows the fibrosis score of Masson-positive area (blue) from ten random fields (original magnification, ×200) of rat kidney samples. Data are represented as the mean±SEM (*n*=6). Means with different letters are significantly different from one another (*P*<0.05). The scale bar in all of the images is 20 μm.

To demonstrate the role of ERK1/2 in uric acid-induced activation of renal interstitial fibroblasts, we created an animal model of hyperuricemic nephropathy. Rats were fed daily with a mixture of adenine and potassium oxonate for 4 weeks. At 0, 7, 14, 21, and 28 days, the kidney was collected to analyze the protein expression of collagen 1 and α-SMA by Western blotting and to assess the fibrotic changes by microscopy after Trichrome Masson Staining. Figure 2A demonstrates that renal expression of collagen 1 and α-SMA was upregulated in a time dependent manner after feeding with adenine and potassium oxonate daily (Figure 2A-2C), with a significant increase at day 7 and maximal expression levels at 21 and 28 days. Consistent with this observation, the kidney sample collected from the rat given adenine and potassium oxonate daily for 7 days also displayed tubular dilation and interstitial expansion with collagen deposition. This was evidenced by an increase in Masson trichrome-positive areas within the tubule interstitium, and each of those changes was more severe at 28 days (Figure 2D).

Periodic acid-Schiff (PAS) staining also showed that the kidneys of hyperuricemic rats displayed severe glomerulosclerosis and renal tubule interstitial injury with tubular dilatation, tubular atrophy and interstitial fibrosis (Supplementary Figure 1) after 0, 7, 14, 21, 28 days of daily feeding with the adenine and potassium oxonate mixture. Serum creatinine and BUN were increased at day 14 and further increased at day 21; the elevated levels were sustained until at least 28 days [9].

**Figure 3:**
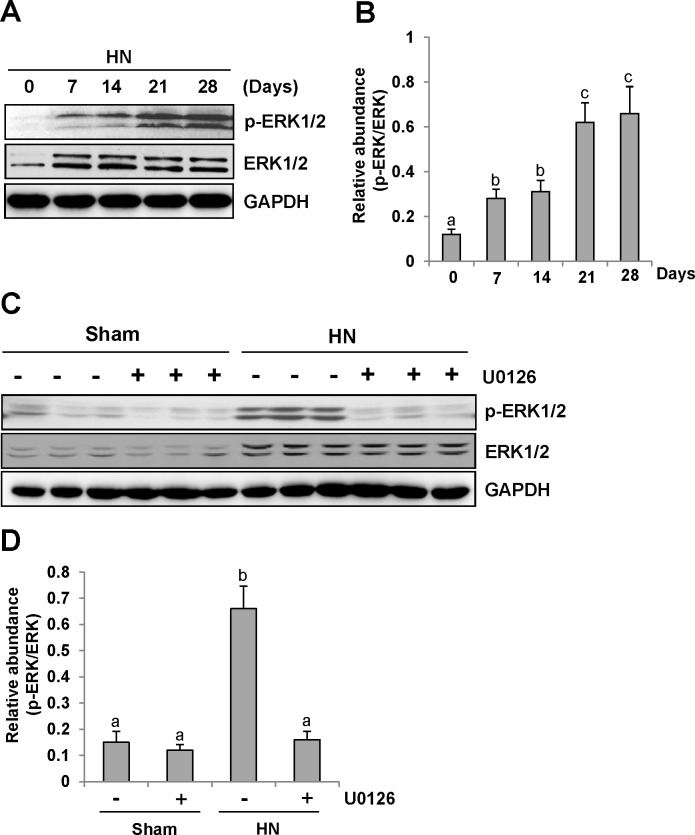
U0126 inhibits ERK1/2 activation in the kidney of hyperuricemic rats **(A)** ERK1/2 was activated in the kidney of HN rats in a time-dependent manner. The kidneys were taken for immunoblot analysis of p-ERK1/2, ERK1/2 or glyceraldehyde 3-phosphate dehydrogenase (GAPDH); the representative results are shown. **(B and D)** Expression level of p-ERK1/2 was quantified by densitometry and normalized with total ERK1/2. **(C)** Rat model of HN was established by feeding with adenine and potassium oxonate daily. In some rats, U0126 were simultaneously administrated intraperitoneally. After 28 days, the kidneys were taken for immunoblot analysis of p-ERK1/2, ERK1/2 or GAPDH. Data are represented as the mean±SEM (*n*=6). Means with different letters are significantly different from one another (*P*<0.05).

Consistent with renal fibrotic changes in the rat model of hyperuricemic nephropathy, we observed increased ERK1/2 phosphorylation in the injured kidney, which occurred at day 7 and remained the same level at day 14, and was further elevated at days 21 and 28 after feeding of the mixture of adenine and potassium oxonate (Figure 3A–3B).

These data clearly suggest that progression of HN is associated with ERK1/2 activation.

### Administration of U0126 blocks ERK1/2 activation in the kidney of hyperuricemic rats

To determine the role of ERK1/2 activation in the progression of HN, we employed U0126, a specific inhibitor of ERK1/2 [32], via I. P. at 10mg/kg every other day in HN rats. As shown in Figure 3C-3D, rats with HN displayed a remarkable up-regulation of ERK1/2 phosphorylation. U0126 treatment decreased the level of phosphorylated ERK1/2 (p-ERK1/2) in the kidney. Densitometry analysis indicates a 92% reduction of p-ERK1/2 in HN rats treated with U0126 compared to those treated with vehicle (Figure 3C, 3D). Although an increase in total ERK1/2 was also observed in the kidney of hyperuricemic rats, their expression levels were not affected by U0126 treatment (Figure 3C–3D). Therefore, we suggest that U0126 is a potent inhibitor of ERK1/2 in HN, providing a powerful tool to examine the functional significance of these kinases in HN.

### U0126 attenuates kidney histopathologic changes and prevents renal dysfunction and proteinuria in hyperuricemic rats

**Figure 4:**
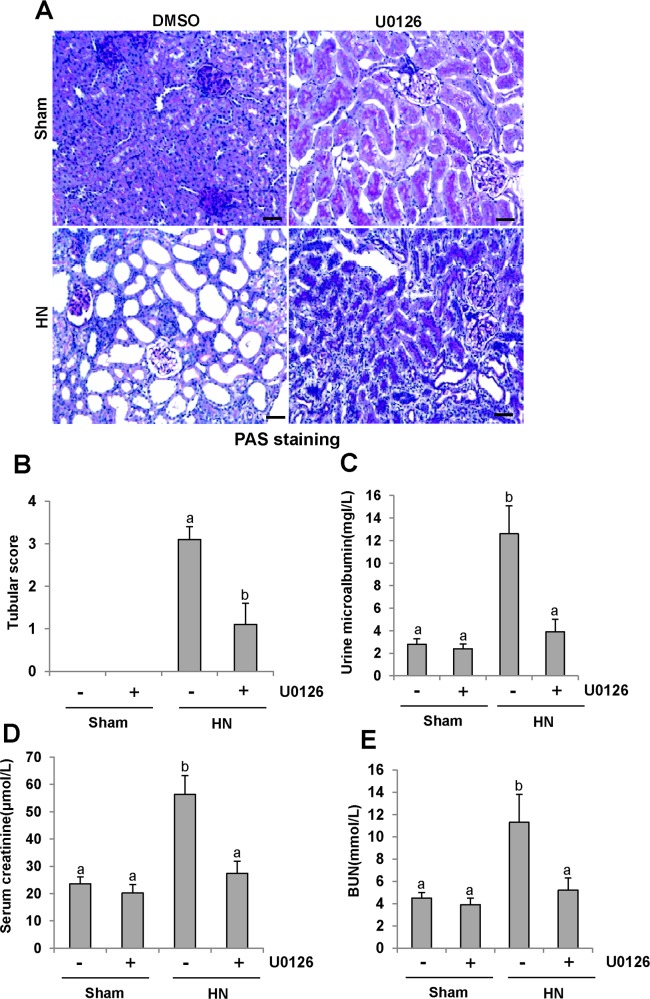
U0126 halts progression of proteinuria and improves renal function and kidney pathology in hyperuricemic rats **(A)** Photomicrographs (original magnification×200) illustrate periodic acid-Schiff staining of the kidney tissues in control or HN rats with or without U0126. **(B)** Morphologic changes were scored on the basis of the scale described in the Concise Methods section. **(C)** Urine microalbumin. **(D)** Expression level of serum creatinine was examined using automatic biochemistry assay. **(E)** Serum BUN. Data are represented as the mean±SEM (*n=*6). Means with different letters are significantly different from one another (*P*<0.05). The scale bar in all of the images is 20 μm.

To determine the role of ERK1/2 activation in HN, we first examined the effect of U0126 on the pathological change of kidneys in hyperuricemic rats. As shown in Figure 4A&4B, inhibition of ERK1/2 preserved kidney architecture and alleviated the glomerular and tubule interstitial damage. Seminal scoring data demonstrated that U0126 improved tubular injury by more than 66.7% (Figure 4B). No significant histopathological changes were observed in the kidney of rats without feeding a mixture of adenine and potassium oxonate (Figure 4A). Next, we assessed the effect of ERK1/2 inhibition on urine microalbumin, serum creatinine, blood urea nitrogen (BUN) and urinary microalbumin/creatinine ratio. As shown in Figure 4C-4E and Supplementary Figure 2, U0126 treatment prevented increases in serum creatinine, BUN, urine microalbumin levels and urinary microalbumin/creatinine ratio in hyperuricemic rats. Collectively, we demonstrated that inhibition of ERK1/2 can improve renal function, reduce proteinuria, and attenuate glomerular and tubular injury in hyperuricemic rats.

### Inhibition of ERK1/2 alleviates the development and progression of renal fibrogenesis in hyperuricemic rats

**Figure 5:**
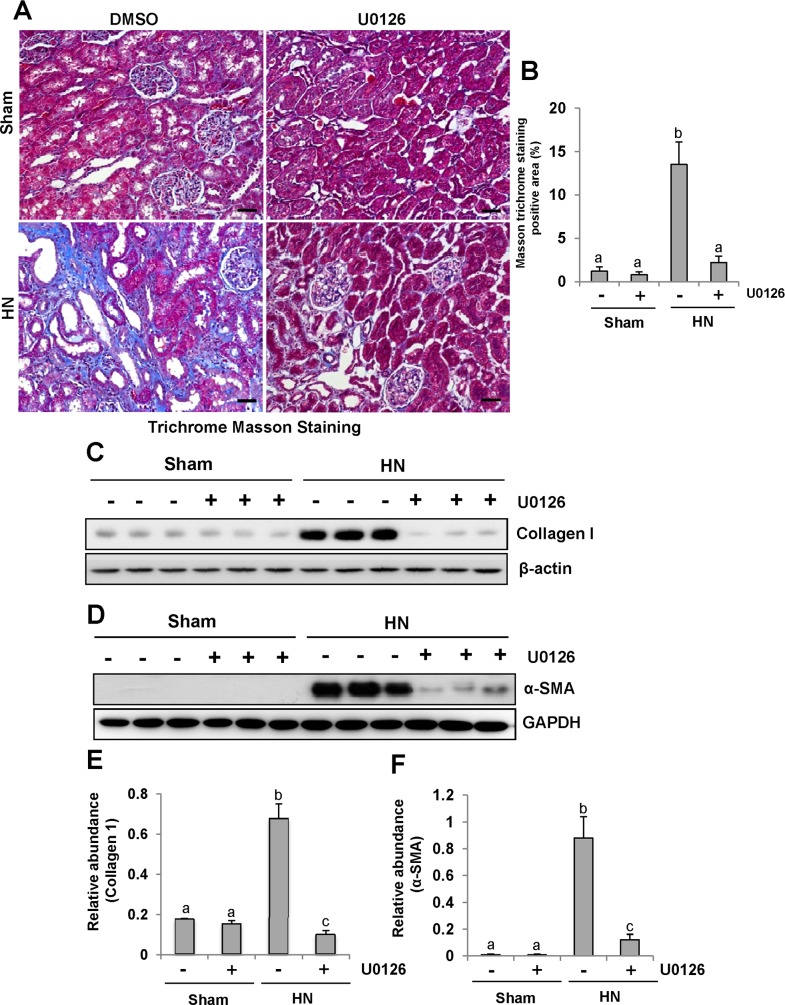
U0126 alleviates the development and progression of renal fibrogenesis in hyperuricemic rats **(A)** Photomicrographs illustrating Masson trichrome staining of kidney tissue collected at day 28 after feeding of mixture of adenine and potassium oxonate with or without U0126. **(B)** The graph shows the percentage of Masson-positive tubulointerstitial area (original magnification, ×200). The kidney tissue lysates were subjected to immunoblot analysis with specific antibodies against collagen I or-β-actin**(C)**, as well as α-SMA or GAPDH **(D)**. **(E)** Expression level of collagen I was quantified by densitometry and normalized with β-actin. **(F)** Expression level of α-SMA was quantified by densitometry and normalized with GAPDH. Data are represented as the mean±SEM (*n=*6). Means with different letters are significantly different from one another (*P*<0.05). The scale bar in all of the images is 20 μm.

Since renal fibrosis is characterized with massive accumulation of extracellular matrix (ECM) proteins in interstitial areas [33–35], we assessed the effect of U0126 on the expression of interstitial collagen fibrils by using Masson trichrome staining. Consistent with our previous data, kidneys of rats given adenine and potassium oxonate oral daily for 3 weeks developed severe morphological damage as evidenced by tubular dilation, epithelial atrophy as well as interstitial expansion with increased collagen accumulation. In contrast, kidneys administrated with U0126 demonstrated a remarkable improvement in the morphological lesions with markedly less fibrosis in the interstitium (Figure 5A-5B). Since collagen I is a major component of the interstitial matrix and α-smooth muscle actin (α-SMA) is the hallmark of activated renal fibroblasts, we also examined the effect of U0126 on their expression. Both collagen I and α-SMA were up-regulated in the kidney of hyperuricemic rats, and administration of U0126 reduced their expression (Figure 5C–5F). Taken together, our results indicate that U0126 is able to inhibit activation of renal interstitial fibroblasts and accumulation of ECM proteins in the kidney of hyperuricemic rats.

### Inhibition of ERK1/2 blocks TGF-β/Smad3 signaling pathways in the kidney of hyperuricemic rats

**Figure 6:**
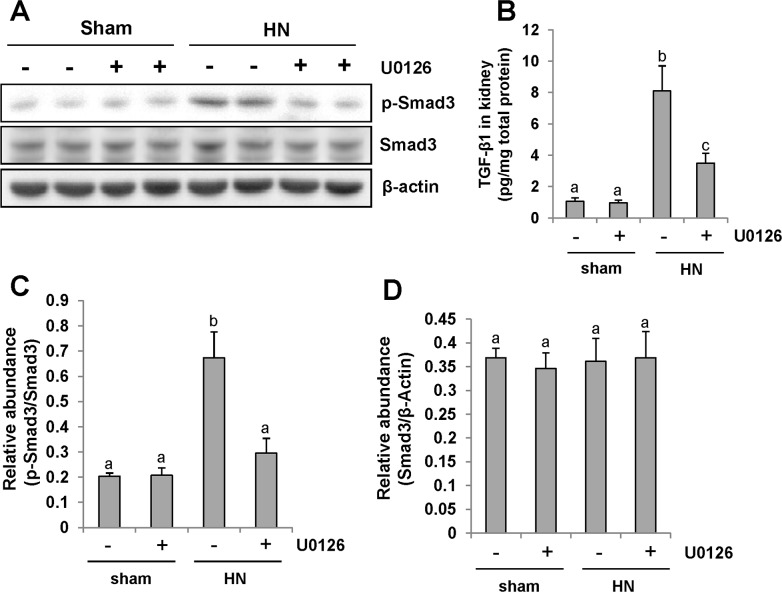
Pharmacologic blockade of ERK1/2 activity suppresses TGF-β1 signaling in the kidney of hyperuricemic rats **(A)** The kidneys were taken from immunoblot analysis of p-Smad3, Smad3, or β-actin. **(B)** Protein was extracted from the kidneys of rats after feeding of the mixture of adenine and potassium oxonate with or without U0126 and subjected to ELISA as described in the Concise Method section. Protein expression level of TGF-β1 was indicated. **(C)** Expression level of p-Smad3 was quantified by densitometry and normalized with total Smad3. **(D)** Expression level of Smad3 was quantified by densitometry and normalized with β-actin. Data are represented as the mean±SEM (*n=*6). Means with different letters are significantly different from one another (*P*<0.05).

ERK1/2 is the downstream signaling molecule of EGFR. Our recent study demonstrated that EGFR modulates uric acid-induced activation of TGF-β signaling [9]. To examine whether ERK1/2 is able to regulate activation of TGF-β/Smad3 signaling, we first examined the level of TGF-β1 in the kidney of HN rats by ELISA. Figure 6B demonstrated that expression of TGF-β1 was significantly increased in the kidney of hyperuricemic rats and administration of U0126 blocked its expression (Figure 6B).

Since Smad3 is the pivotal downstream mediator of TGF-β signaling and mediates the transcription of TGF-β targeted fibrotic associated genes [36], we also compared the level of phosphorylated Smad3 (p-Smad3) in the kidney of hyperuricemic rats treated or untreated with U0126. Western blot analysis of kidney lysates indicated that HN injury-induced phosphorylation of Smad3 was significantly inhibited by U0126 as shown in Figure 6A, 6C. Expression of total smad3 was not affected by HN injury and U0126 treatment (Figure 6A, 6D). Collectively, our results suggest that U0126 is able to inhibit activation of TGF-β signaling in hyperuricemia associated kidney diseases.

### ERK1/2 inhibition abrogates NF-*κ*B(p65) phosphorylation in the kidney of hyperuricemic rats

**Figure 7:**
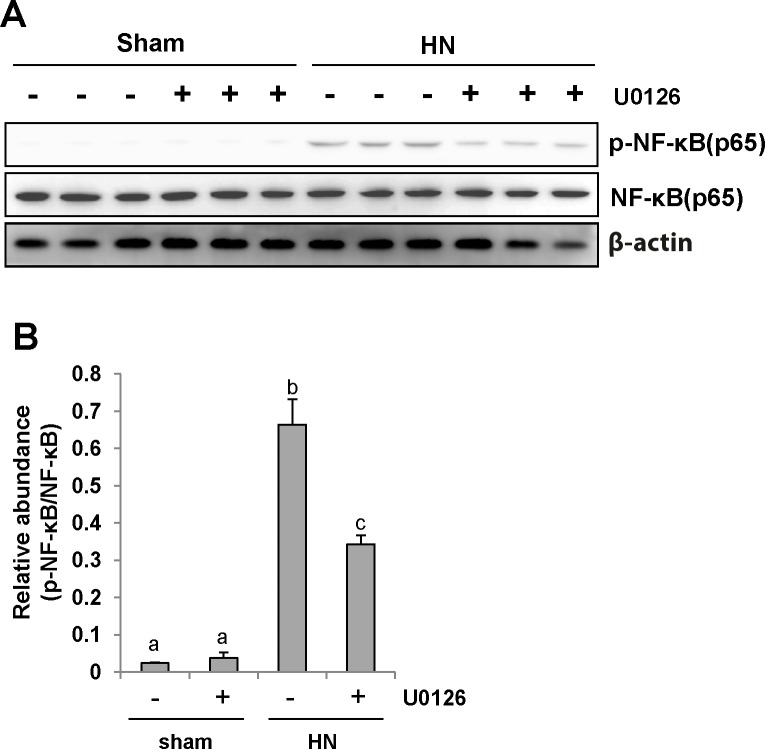
U0126 inhibits NF-*κ*B pathway activation in the kidney of hyperuricemic rats **(A)** The kidney tissue lysates were subjected to immunoblot analysis with specific antibodies against p-NF-*κ*B(p65), NF-*κ*B (p65), or β-actin. **(B)** Expression level of p-NF-*κ*B (p65) was quantified by densitometry and normalized with NF-*κ*B (p65). Data are represented as the mean±SEM (*n=*6). Means with different letters are significantly different from one another (*P*<0.05).

NF-*κ*B is a transcriptional factor associated with chemokine expression regulation, and its activation is a consequence of the inflammatory responses. Immunoblot analysis of the kidney lysates indicated that expression of phosphorylated NF-κB (p-NF-κB p65) was upregulated in hyperuricemic rats (Figure 7A and 7B) and significantly downregulated by inhibiting ERK1/2. Phosphorylated NF-*κ*B was scarcely detectable in the sham group with or without U0126. The level of total NF-*κ*B was unchanged for each group of rats (Figure 7A). Therefore, these data indicate that activation of ERK1/2 contributes to the NF-*κ*B signaling pathway activation in hyperuricemia-induced kidney diseases.

### U0126 inhibits expression of cytokines in the kidney of hyperuricemic rats

**Figure 8:**
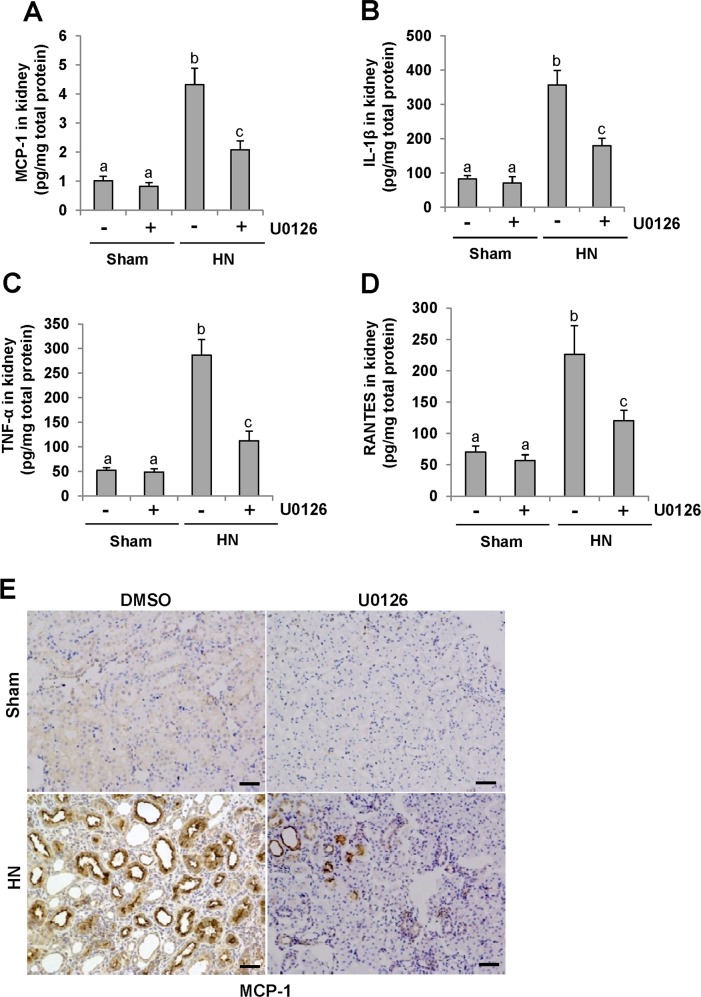
U0126 reduces the expression of MCP-1, IL-1β, TNF-α and RANTES in the kidney of hyperuricemic rats **(A)** Protein was extracted from the kidneys of rats after feeding of the mixture of adenine and potassium oxonate with or without U0126 treatment and subjected to ELISA as described in the Concise Methods section. Protein expression level of MCP-1 was indicated. **(B)** IL-1β. **(C)** TNF-α. **(D)** RANTES. **(E)** Photomicrographs (original magnification ×200) illustrate MCP-1 staining of the kidney tissues. Data are represented as the mean±SEM (*n=*6). Means with different letters are significantly different from one another (*P*<0.05). The scale bar in all of the images is 20 μm.

It has been demonstrated that proinflammatory cytokines such as MCP-1, TNF-α, IL-1β and RANTES are significantly increased in the fibrotic kidney [37] and the inflammatory response is the initial and key step in renal fibrogenesis [38]. To examine the effect of U0126 on the expression of these cytokines, we measured their expression levels in the hyperuricemic kidney treated or untreated with U0126 using ELISA. As demonstrated in Figure 8A-8D, all of these cytokines were increased in the kidneys of rats given adenine and potassium oxonate daily for 3 weeks; treatment with U0126 significantly downregulated expression levels of MCP-1, TNF-α, IL-1β and RANTES. Further immunochemical investigation also demonstrated that U0126 remarkably decreased expression of MCP-1 in the kidney of hyperuricemic rats (Figure 8E). MCP-1 was highly expressed in injured renal tubules of hyperuricemia rats. Thus, these data suggest that blockade of ERK1/2 inhibited expression of proinflammatory factors in hyperuricemic rats.

### U0126 inhibits macrophage infiltration in the kidney of hyperuricemic rats

**Figure 9:**
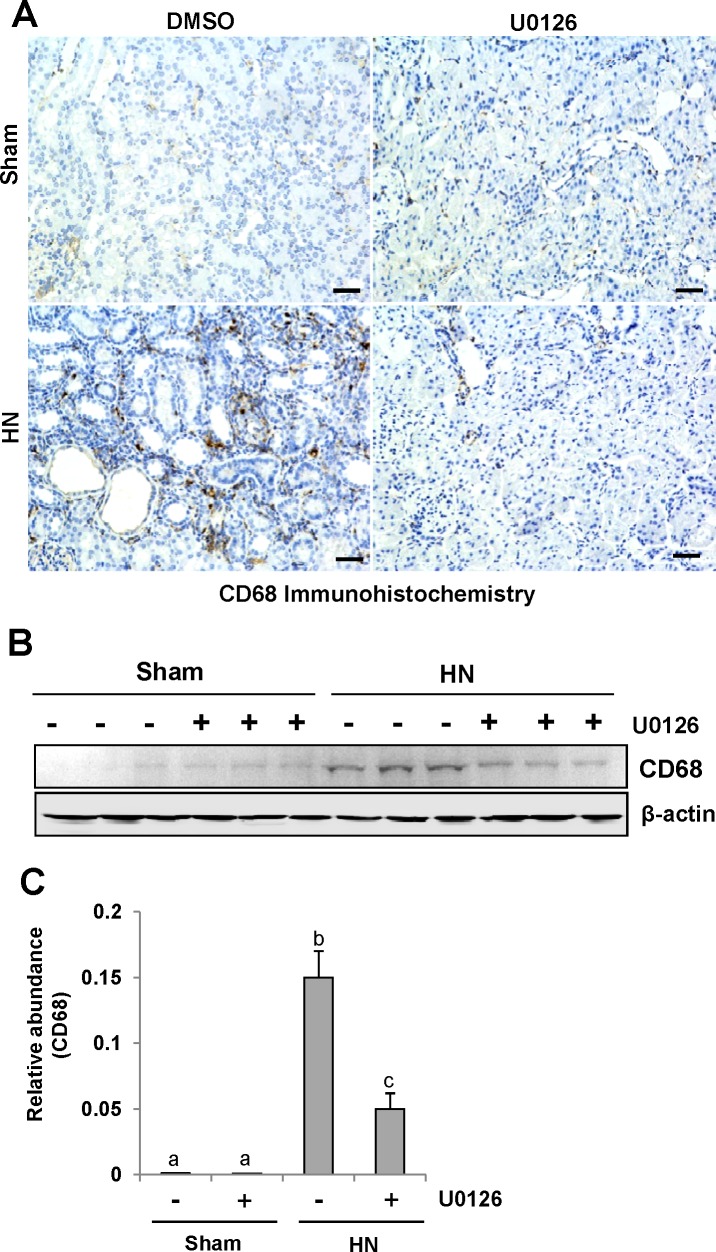
U0126 inhibits macrophage infiltration in the kidney of hyperuricemic rats **(A)** Photomicrographs (original magnification ×200) illustrate CD68 staining of the kidney tissues. **(B)** The kidney tissue lysates were subjected to immunoblot analysis with specific antibodies against CD68 or β-actin. **(C)** Expression level of CD68 was quantified by densitometry and normalized with β-actin. Data are represented as the mean±SEM (*n=*6). Means with different letters are significantly different from one another (*P*<0.05). The scale bar in all of the images is 20 μm.

CD68 is a well-known biomarker of macrophage infiltration [39]. To assess the role of U0126 on macrophage infiltration, we examined the localization and expression of CD68 by immunohistochemistry and immunoblot analysis. As shown in Figure 9 A, the number of infiltrated macrophage remarkably increased in the renal interstitium of HN rats, and was dramatically decreased after treatment with U0126. Immunoblot analysis also demonstrated a reduction in the expression of CD68 in the kidney of HN rats (Figure 9, 9B-9C). Thus, these data indicate that inhibition of ERK1/2 activation effectively inhibits the accumulation of macrophages in the kidney of hyperuricemic rats.

### ERK1/2 inhibition reduces serum uric acid and XOD activity, prevents a rise of URAT1 and down regulates OAT1 and OAT3

**Figure 10:**
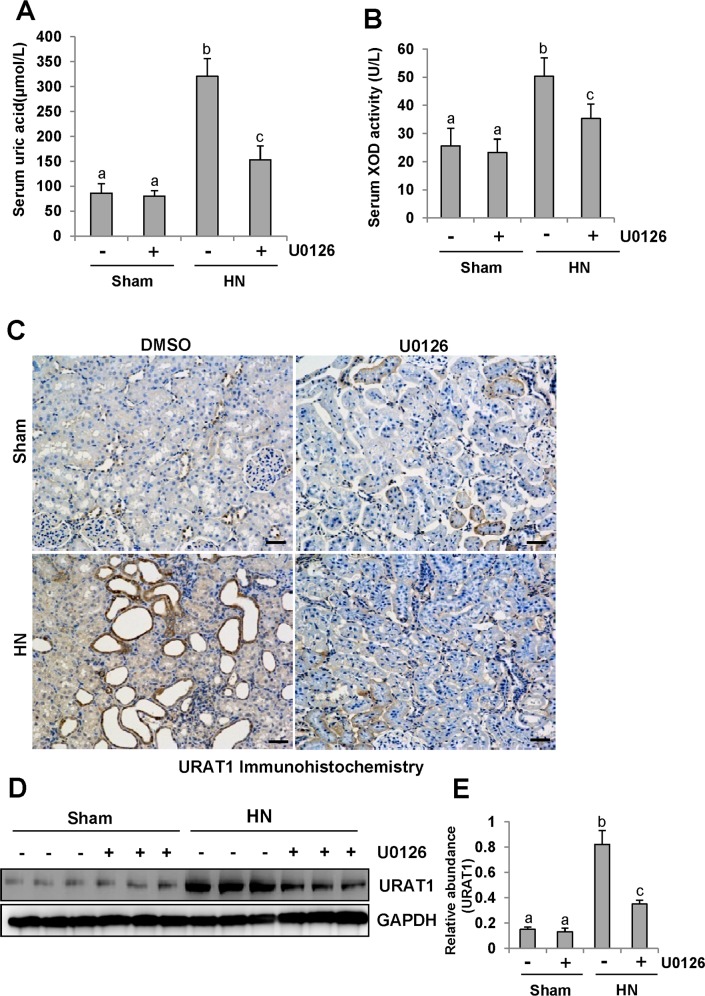
ERK1/2 inhibition reduced serum uric acid and XOD activity and preserved the expression of one key urate transporters **(A)** Expression level of serum uric acid was examined using automatic biochemistry assay (P800; Modular). **(B)** Serum XOD activity was examined by XOD kit. **(C)** Photomicrographs (original magnification ×200) illustrate URAT1 staining of the kidney tissues. **(D)** The kidney tissue lysates were subjected to immunoblot analysis with specific antibodies against URAT1 or glyceraldehyde 3-phosphate dehydrogenase (GAPDH). **(E)** Expression level of URAT1 was quantified by densitometry and normalized with GAPDH. Data are represented as the mean±SEM (*n=*6). Means with different letters are significantly different from one another (*P*<0.05). The scale bar in all of the images is 20 μm.

Research has elucidated that hyperuricemia is tightly linked with increased XOD activity [40]. Thus, we evaluated the effect of U0126 on the production of uric acid and the activity of serum XOD in HN rats. After three weeks of daily feeding of the mixture of adenine and potassium oxonate, serum uric acid levels were increased three fold above levels in rats that were not fed the mixture as indicated in Figure 10A. Inhibition of ERK1/2 dramatically decreased serum uric acid levels(Figure 10A). Figure 10B demonstrates that the activity of serum XOD nearly doubled in the kidney of hyperuricemic rats compared with rats that were not fed the adenine and potassium oxonate mixture. U0126 remarkably reduced the activity of serum XOD in hyperuricemic rats.

To understand the mechanisms of how U0126 regulates the metabolism of serum uric acid, we determined the localization and expression of URAT1 by immunohistochemistry and western blotting. As indicated in Figure 10C, URAT1 was mostly expressed in injured tubules of hyperuricemic rats. Administration of U0126 reduced its expression (Figure 10C–10E).

**Figure 11:**
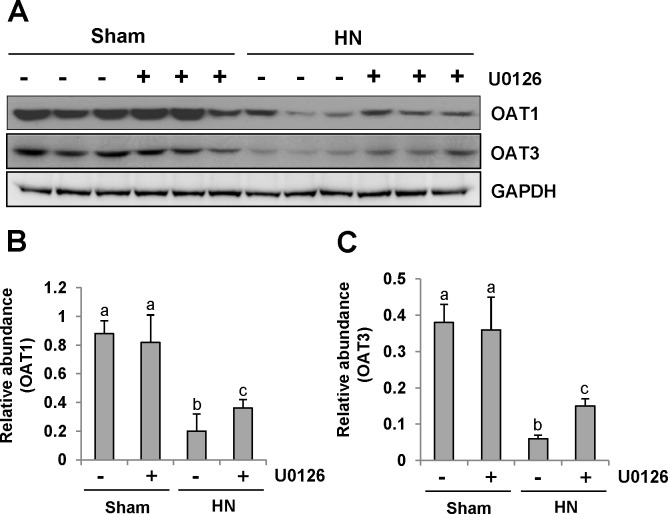
U0126 administration preserves the expression of two key urate transporters **(A)** The kidney tissue lysates were subjected to immunoblot analysis with specific antibodies against OAT1, OAT3 or glyceraldehyde 3-phosphate dehydrogenase (GAPDH). **(B)** Expression level of OAT1 was quantified by densitometry and normalized with GAPDH. **(C)** Expression level of OAT3 was quantified by densitometry and normalized with GAPDH. Data are represented as the mean±SEM (*n=*6). Means with different letters are significantly different from one another (*P*<0.05).

Since human urate transporters such as OAT1 and OAT3 have been shown to be critically involved in uric acid homeostasis [41], we assessed the effect of ERK1/2 inhibition on their expression in the kidney of hyperuricemic rats. As shown in Figure 11A, an abundance of OAT1 and OAT3 was detected in the sham-treated kidney, and their expression levels were dramatically reduced in the hyperuricemic kidney. Administration of U0126 partially preserved expression of OAT1 and OAT3 in the hyperuricemic kidney(Figure 11A-11C).

These data suggests that ERK1/2 activation plays a distinct role in the regulation of URAT1, OAT1 and OAT3. It also effectively enhances the expression of URAT1 and decreases the expression of OAT1 and OAT3 in the hyperuricemic kidney.

## DISCUSSION

Hyperuricemic nephropathy (HN) has been considered as a key component in the transition of the CKD population into ESRD [42]. In this study, we examined the role of ERK1/2 in the pathogenesis of HN in a rat model using a specific ERK1/2 inhibitor, U0126. Our results showed that ERK1/2 were activated in the kidney of hyperuricemic nephropathy and pharmacological blockade of ERK1/2 activation with U0126 improved renal function, decreased urine microalbumin, attenuated pathological changes, inhibited activation of renal interstitial fibroblasts and decreased accumulation of extracellular matrix proteins in the hyperuricemic kidney. Furthermore, inhibition of ERK1/2 with U0126 or siRNA reduced uric acid-induced expression of α-SMA and collagen I in cultured renal interstitial fibroblasts. These results indicate that the ERK1/2 pathway is critically involved in the activation of renal fibroblasts and pathogenesis of hyperuricemic nephropathy, and suggest that inhibition of this signaling pathway may be a potential approach for prevention and treatment of hyperuricemic nephropathy.

Currently, four methods have been documented in the literature outlining methods of establishing HN animal models, including use of a urate oxidase inhibitor [43–45], purine-rich diets [46], administration of a mixture of adenine and potassium oxonate [47–50] and disruption of the Uox gene leading to uricase deficiency in mice [51–53]. Among them, administration of a mixture of adenine and potassium oxonate is the most common and effective method to establish the model of hyperuricemic nephropathy [47–50]. As a result, we choose this model. The mechanism by which ERK1/2 inhibition attenuates HN remains incompletely explored. Several potential mechanisms may contribute to the protective effect of ERK1/2 inhibition on hyperuricemic nephropathy. First, ERK1/2 inhibition leads to suppression of the TGF-β signaling. It is well known that TGF-β signaling pathway plays a key role in the development of renal interstitial fibrosis [30], and its inhibition by different strategies significantly alleviates fibrotic lesions in the kidney and improves renal injury [54]. In this pathway, Smad3 acts downstream of TGF-β receptors to induce the transcriptional expression of numerous profibrotic genes such as collagen1 and fibronectin [30, 54]. Interestingly, there are also reports showing that ERK1/2 can directly induce phosphorylation of Smad3 and enforce its function to transducer profibrotic signals [55]. Our results indeed indicate that inhibition of ERK1/2 not only significantly suppressed expression of TGF-β1, but also blocked phosphorylation of Smad3 in HN rats and in cultured renal fibroblasts, suggesting the importance of the TGF-β1/Smad3 signaling pathway in mediation of ERK1/2–induced HN. Consistent with our observations, Kim et al., also showed that uric acid-induced up-regulation of TGF-β1 depends on the activation of ERK1/2 signaling pathway in diabetic kidneys [56].

Second, ERK1/2 inhibition may protect against hyperuricemic nephropathy through inhibition of proinflammatory response. An early study showed that mild hyperuricemia has vasoactive and proinflammatory effects independent of crystal formation induced renal injury [18] whereas treatment with rasburicase (Recombinant urate oxidase) reversed the inflammatory damages and lessened tubular injury with an improvement in renal function [18]. Hyperuricemia can cause marked tubular injury, up-regulated expression of MCP-1 and macrophage infiltration without intra renal crystals in the kidney of hyperuricemic rats [9]. In this study, we demonstrated that blocking ERK1/2 activation reduced renal expression of multiple cytokines/chemokines including MCP-1, TNF-α, IL-1β and RANTES, and attenuated infiltration of macrophages to the injured kidney. Given that those proinflammatory changes contributes to acute and chronic renal injury in various animal models of kidney diseases [57], ERK1/2 inhibition-mediated suppression of proinflammatory response may contribute to alleviation of renal injury and improvement in renal function.

Third, ERK1/2 inhibition mediated renal protection may be involved in the regulation of serum uric acid levels. This is evident by our observations that U0126 significantly decreased serum uric acid levels. Uric acid is the final product of purine metabolism in humans and serum uric acid levels are determined by the balance of uric acid synthesis and renal excretion [58]. Hyperuricemia depends on increased production, decreased excretion of uric acid, or a combination of these two mechanisms. Since enzyme xanthine oxidase (XO) is a fundamental enzyme which promotes production of serum uric acid [58], we first examined the effect of U0126 on the expression of XO and found that inhibition of ERK1/2 suppressed XO activity. Given that OAT1 and OAT3 are two primary organic anion transporters that mediate excretion of uric acid to the tubular lumen and their aberrant expression causes excessive uric acid accumulated in the human body, leading to hyperuricemia [58, 59], we also examined the effect of U0126 on their expression. Our results demonstrated that expression levels of both OAT1 and OAT3 were reduced in HN injured kidneys and partially resumed when ERK1/2 pathways were inhibited. On this basis, we suggest that the ERK1/2 pathway is critically involved in the up-regulation of uric acid levels via increasing the XO activity and reducing expression of OAT1 and OAT3.

However, ERK1/2 inhibition may also reduce serum uric acid levels through interfering with the cellular and molecular machinery associated with the reabsorption of uric acid in the kidney. In the human kidney, filtered uric acid is mainly reabsorbed in the proximal tubules [58] and regulated by a urate reabsorption transporter known as renal urate transporter 1 (URAT1). URAT1 is expressed in the apical membrane of proximal tubule cells by the SLC22A12 gene, and mutation of the gene causes idiopathic renal hypouricemia [60, 61]. As such, we also examined the expression of URAT1 in the injured kidney and the effect of ERK1/2 inhibition. Our results revealed that expression levels of URAT1 were increased in the rat kidney with HN alone and ERK1/2 inhibition blocked this response. This suggests that ERK1/2 activation also promotes the reabsorption of uric acid by enhancing URAT1 expression. As such, we suggest that the ERK1/2 inhibitor elicited up-regulation of URAT1 expression may also contribute to the homeostasis of uric acid in hyperuricemic nephropathy.

Although our findings indicate that ERK1/2 activation is critically implicated in the regulation of serum uric acid levels and molecular mechanisms involved, it should be noted that inhibition of U0126-elicited regulation on those pathological changes was incomplete. This suggests that in addition to ERK1/2, other signaling mechanisms also contribute to these processes. MAPK pathways are composed of ERK1/2, Jun-NH2-terminal kinase (JNK), and p38 pathways [62]. Upon activation, these kinases are phosphorylated and subsequently translocated to nuclei where they trigger activation of multiple transcription factors including Elk1, Jun, fos. A large number of studies have indicated that MAPKs are pivotal mediators in pathophysiology of kidney insults [63]. Currently, it remains unclear whether JNK and p38 pathways also participate in regulating the homeostasis of uric acid and pathogenesis of hyperuricemic nephropathy. Future studies are required to examine their role in these processes.

It has been documented that an elevated serum uric acid independently predicts the development of CKD [64]. Inhibition of increased serum uric acid can protect from renal function impairment in some cases [9, 64]. Experimental investigations have indicated the key role of uric acid not only in CKD, but also in acute kidney injury, hypertension, diabetes, and metabolic syndrome [4, 5, 64]. Pilot studies suggest that lowering plasma uric acid concentrations may slow the progression of renal disease in subjects with CKD [65]. However, the mechanism of hyperuricemia-induced nephropathy remains largely unknown. Our study not only provides the evidence that ERK1/2 are important mediators in HN, but also suggests that they are potential targets for treatment of this disease.

In summary, our results demonstrate that inhibition of ERK1/2 markedly decreases serum uric acid levels and alleviates development and progression of HN through multiple mechanisms, including suppression of pre-inflammatory cytokines, blockade of TGF-β1/Smad3 signaling and lowering serum uric acid levels via modulating expression of XOD and urate transporters. Therefore, ERK1/2 activation may lay down an important molecular basis for hyperuricemia-mediated renal injury and pharmacological targeting of this pathway may be a potential therapeutic treatment for HN.

## MATERIALS AND METHODS

### Chemicals and Antibodies

Antibodies to p-ERK1/2, ERK1/2, p-Smad3, Smad3 were purchased from Cell Signaling Technology (Danvers, MA). Antibodies to MCP-1, OAT1, OAT3, fibronectin, collagen 1(A2), GAPDH, β-actin, EGFR and CD68, siRNA were purchased from Santa Cruz Biotechnology, Inc. (Santa Cruz, CA). U0126 was purchased from Med Chem Express (Monmouth Junction, NJ). Serum XOD kit was from JianchengTechnology (Nanjing, China). MCP-1, TNF-α, IL-1β, RANTES and TGF-β1 enzyme-linked immunosorbent assay (ELISA) kits were from R&D systems (Minneapolis, MN). Vectastain ABC kit was from Vector Laboratories (Burlingame, CA). Antibodies to α-SMA, DMSO, and all other chemicals were from Sigma (St. Louis, MO).

### Cell culture and treatments

Rat renal interstitial fibroblasts (NRK-49F) were cultured in Dulbecco's modified eagle's medium (DMEM) (Sigma-Aldrich, St. Louis, MO) containing 5% fetal bovine serum (FBS), 0.5% penicillin and streptomycin in an atmosphere of 5% CO_2_ and 95% air at 37°C. To examine the role of ERK1/2 in uric acid-induced renal fibroblast activation, NRK-49F were starved with 0.5% FBS for 24h and then exposed to various concentrations of uric acid (0-800 μM) for 36 h. Then, cells were harvested for immunoblot analysis.

### siRNA transfection

The small interfering (si) RNA oligonucleotides targeted specially for ERK1/2 was used in this study. NRK-49F were seeded to 30-40% confluence in antibiotic-free medium, and then incubated for 24 h prior to ERK1/2 siRNA (750pmol) transfection with lipofectamine 2000. In parallel, scrambled siRNA (750 pmol) was used as control for off-target changes in NRK-49F. After transfection, cells were cultured in DMEM-F12 treated with uric acid (800 μM) for an additional 36h before being used for the experiments. Then, cells were harvested for immunoblot analysis.

### Hyperuricemic nephropathy (HN) rats model and U0126 treatment

Male Sprague-Dawley rats (6-8 weeks old) that weighed 200-220 g were purchased from B&K laboratory animal Corp (Shanghai, China). 24 male rats were randomly assigned to 4 groups of six rats: sham, sham treated with ERK1/2, HN, and HN treated with ERK1/2. The HN rat model was established as described in our previous study [9]. Briefly, a mixture of adenine (0.1g/kg) and potassium oxonate (1.5g/kg) dissolved in distilled water was administered via P.O. once daily for three weeks. To examine the effect of U0126 on renal protection in HN rats, U0126 (10 mg/kg) in 50 μl of dimethyl sulfoxide (DMSO) was administrated via peritoneal injection every other day to the rats 1 hour after the mixture of adenine and potassium oxonate exposure was taken by rats. For the HN alone group, rats were injected with an equivalent amount of DMSO. At day 21, the animals were sacrificed and the kidneys were collected for protein analysis and histological examination. 24-hour urine samples were collected in metabolic cages at day 0 and weekly for determination of urinary proteins. In addition, blood was also taken once a week for the measurement of serum uric acid, BUN, creatinine, and other biochemistry index as indicated in our previous report [9]. For the time course study, blood, urine and kidneys were collected at 0, 7, 14, 21, 28 days after daily feeding of the mixture of adenine and potassium oxonate, and all the indicated parameters were determined.

### Assessment of serum uric acid, renal Function and other biochemistry index

Serum creatinine, uric acid, and BUN, urinary microalbumin, urinary uric acid excretion were examined via automatic biochemistry assay (P800, Modular, USA) as indicated in our previous study [9]. Collected blood was centrifuged at 2500RPM/min for 5min and 200μl serum was collected and put in an automatic biochemistry analyzer (P800, Modular, USA) for analysis.

### Analysis of serum activity of XOD

Serum activity of Xanthine oxidase (XOD) was determined according to the protocol provided by the manufacture (20100818, Jiancheng, Nanjing, china).

### Immunoblot analysis

Immunoblot analysis of NRK-49F cells and tissue samples were conducted as described previously [66]. The densitometry analysis of immunoblot results was conducted by using NIH Image software (National Institutes of Health, Bethesda, MD).

### Immunohistochemical staining

As previously [42], renal tissue was fixed in 4.5% buffered formalin, dehydrated and embedded in paraffin. For general histology, sections were stained with PAS. Immunohistochemical staining was performed based on the procedure described in our previous studies [66]. Masson trichrome staining was conducted according to the protocol provided by the manufacture (Sigma, St. Louis, MO). The collagen tissue area (blue color) was quantitatively measured using Image Pro-Plus software (Media-Cybernetics, Silver Spring, MD, USA) by drawing a line around the perimeter of positive staining area, and the average ratio to each microscopic field (400X) was calculated and graphed. To evaluate the extent of tubular injury, morphological damage (epithelial necrosis, luminal necrotic debris, and tubular dilation) in 3–4 sections per kidney and 10–12 fields per section were quantified using the following scale: none=0; <10%=1; 11–25%=2; 26–75%=3; and >75%=4). Severity of inflammation was graded by counting the absolute number of CD68–positive and MCP-1 positive cells in each field and reported as the mean of 20 random high-power (x400) fields each rat in six rats per group.

### ELISA analysis

To determine the expression of TGF-β1, RANTES, MCP-1, TNF-α and IL-1β, rat kidneys were homogenized in lysis buffer, and the supernatant was recovered after centrifugation. Cytokines’ level of renal tissue was determined by using the commercial Quantikine ELISA kit in accordance with the protocol specified by the manufacturer (ELISA kit, R&D systems, Minneapolis, MN) as reported previously [9]. Total protein levels were examined by using a bicinconinic acid protein assay kit. The concentration of cytokines in kidneys was expressed as pico grams per milligram of total proteins.

### Statistical analysis

All the experiments were performed at least three times. Data depicted in graphs represent the means ± SEM for each group. Inter-group comparisons were made using one-way analysis of variance (ANOVA). Multiple means were compared using Tukey's test. The differences between two groups were determined by Student t-test. Statistical significant difference between mean values was marked in each graph. P<0.05 is considered as significant difference.

## SUPPLEMENTARY MATERIALS FIGURES AND TABLES


