# Supplementary material for: Pharmacologic targeting ERK1/2 attenuates the development and progression of hyperuricemic nephropathy in rats
Source: Oncotarget. 2017 Apr 10;8(20):33807–26. doi: 10.18632/oncotarget.16995 (PMC5464913; doi:10.18632/oncotarget.16995)
Supplement: Supplementary file 1 [file oncotarget-08-33807-s001.pdf]

Pharmacologic targeting ERK1/2 attenuates the development and progression of hyperuricemic nephropathy in rats

SUPPLEMENTARY FIGURES

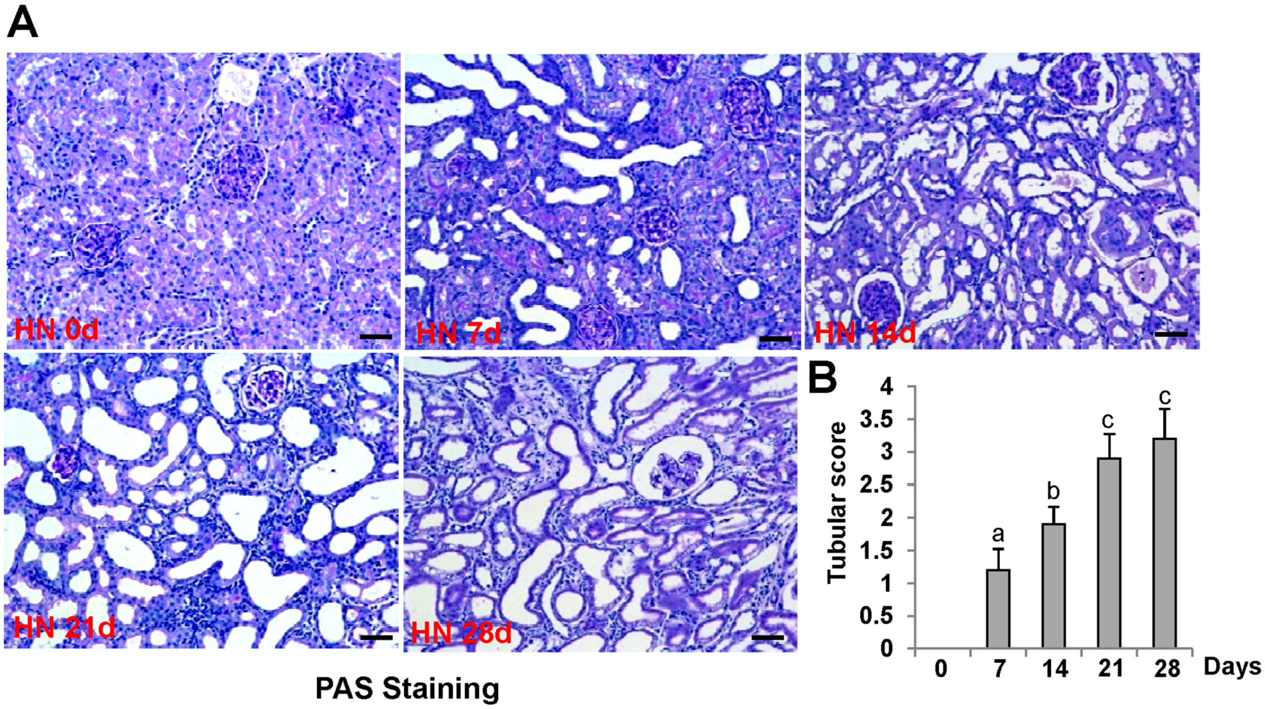

Supplementary Figure 1: Pathological changes in the kidney of hyperuricemic rats in a time-dependent manner. (A) Photomicrographs (original magnification, ×200) illustrate periodic acid-Schiff staining of the kidney tissues of hyperuricemic rats in a time-dependent manner. **(B)** Morphologic changes were scored on the basis of the scale described in the Concise Methods section. Data are represented as the mean±SEM (n=6). Means with different letters are significantly different from one another (P<0.05).The scale bar in all of the images is 20 μm.

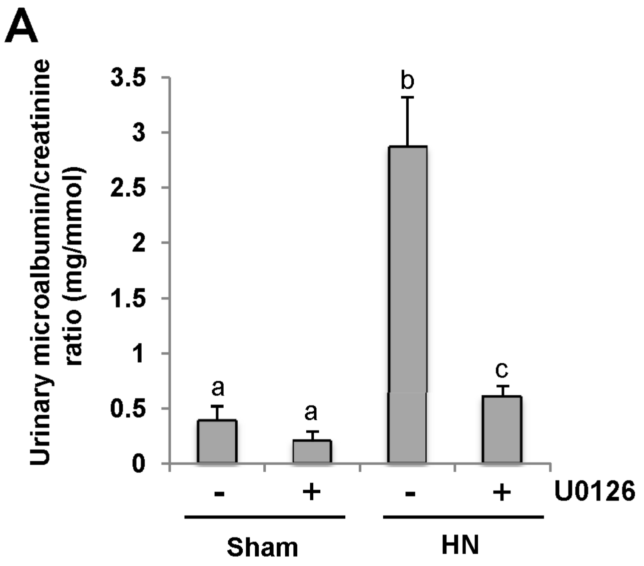

Supplementary Figure 2: U0126 alleviates urinary microalbumin/creatinine ratio in hyperuricemic rats. Urinary microalbumin and creatinine were examined using automatic biochemistry assay. The microalbumin/creatinine ratio was calculated. Data are represented as the mean±SEM (*n*=6). Means with different letters are significantly different from one another ($P<0.05$).
